# How motivation affects academic performance: a structural equation modelling analysis

**DOI:** 10.1007/s10459-012-9354-3

**Published:** 2012-02-22

**Authors:** R. A. Kusurkar, Th. J. Ten Cate, C. M. P. Vos, P. Westers, G. Croiset

**Affiliations:** 1 Center for Research and Development of Education, University Medical Center Utrecht, P.O. Box # 85500, 3508 GA Utrecht, The Netherlands; 2 Rudolf Magnus Institute of Neuroscience, University Medical Center Utrecht, Utrecht, The Netherlands; 3 Institute for Research and Education, VU University Medical Center Amsterdam, Amsterdam, The Netherlands; 4 Department of Biostatistics, Julius Center for Health Sciences and Primary Care, University Medical Center Utrecht, Amsterdam, The Netherlands

**Keywords:** Autonomous motivation, Controlled motivation, Study strategy, Study effort, Academic performance, Self-determination theory

## Abstract

Few studies in medical education have studied effect of quality of motivation on performance. Self-Determination Theory based on quality of motivation differentiates between Autonomous Motivation (AM) that originates within an individual and Controlled Motivation (CM) that originates from external sources. To determine whether Relative Autonomous Motivation (RAM, a measure of the balance between AM and CM) affects academic performance through good study strategy and higher study effort and compare this model between subgroups: males and females; students selected via two different systems namely qualitative and weighted lottery selection. Data on motivation, study strategy and effort was collected from 383 medical students of VU University Medical Center Amsterdam and their academic performance results were obtained from the student administration. Structural Equation Modelling analysis technique was used to test a hypothesized model in which high RAM would positively affect Good Study Strategy (GSS) and study effort, which in turn would positively affect academic performance in the form of grade point averages. This model fit well with the data, Chi square = 1.095, *df* = 3, *p* = 0.778, RMSEA model fit = 0.000. This model also fitted well for all tested subgroups of students. Differences were found in the strength of relationships between the variables for the different subgroups as expected. In conclusion, RAM positively correlated with academic performance through deep strategy towards study and higher study effort. This model seems valid in medical education in subgroups such as males, females, students selected by qualitative and weighted lottery selection.

## Introduction

Motivation has been shown to positively influence study strategy, academic performance, adjustment and well-being in students in domains of education other than medical education (Vansteenkiste et al. 2005). Studying motivation particularly in medical students is important because medical education is different from general education in several aspects, some of them being high intensity of study, the requirement to carry out clinical work along with study and the need to follow a highly specifically defined path to be able to qualify to practice as doctors. In a literature review we found that the positive correlation between motivation and performance has not been substantiated strongly in medical education as different studies have contradictory findings (Kusurkar et al. 2011). The objective of the present research study was to explore the relationships between motivation, study strategy, study effort and academic performance among medical students.

**Fig. 1:**
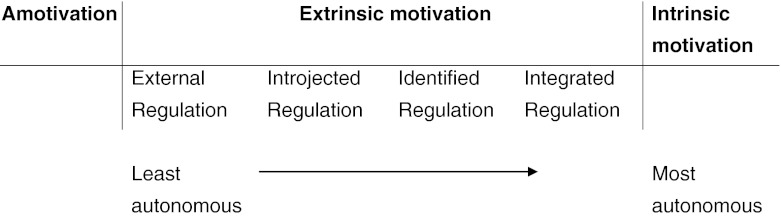
The self-determination continuum (adapted from Deci and Ryan 2000)

There are different theories of motivation; some focus on quantity of motivation and others on quality. Quantity of motivation could be high or low. Quality of motivation depends on whether the source of motivation is internal or external. Self-determination Theory (SDT) of motivation considers quality of motivation to be more important than quantity and describes a continuum for quality of motivation (Ryan and Deci 2000a, b). This ranges from intrinsic motivation at one end to amotivation at the other end of the continuum, with four types of extrinsic motivation (integrated regulation, identified regulation, introjected regulation, external regulation) in between. Intrinsic motivation is derived out of genuine interest in an activity. Extrinsic motivation is derived out of an expected gain or a separable outcome. As elaborated by SDT, not all types of extrinsic motivation are undesirable. Extrinsic motivation spans from high self-determination to low self-determination (see Fig. 1; Ryan and Deci 2000a, b). Identified Regulation, the highly autonomous type of extrinsic motivation, is close to intrinsic motivation. Identified regulation and intrinsic motivation can be summed up to generate Autonomous Motivation (AM). Thus AM depicts self-determined motivation. Introjected and external regulation, which are low in self-determination, can be summed up together to generate Controlled Motivation (CM). Thus CM depicts motivation which is very low on self-determination.

SDT advocates that the more self-determined or autonomous the motivation, the better are the observed outcomes (Ryan and Deci 2000a, b): namely deep learning (Vansteenkiste et al. 2005; Grolnick and Ryan 1987), high academic performance (Soenens and Vansteenkiste 2005; Boggiano et al. 1993), better adjustment and positive well-being (Black and Deci 2000; Levesque et al. 2004).

In the present study we measured Autonomous Motivation (AM) and Controlled Motivation (CM) as described by SDT (Vansteenkiste et al. 2005; Grolnick and Ryan 1987).

Motivation has been reported in primary, secondary and college education to influence academic performance through study effort as a mediator (Vansteenkiste et al. 2005). This relationship, to our knowledge, has never been tested in medical education. We searched for articles in medical education employing Structural Equation Modelling (SEM) as a methodology and found articles studying factors leading to choice of specialty in medicine (Williams et al. 1994, 1997), basic science and clinical knowledge (Schmidt and Moust 1995), clinical reasoning (De Bruin et al. 2005), use of SEM in medical education (Violato and Hecker 2007), influence of clerkships on student learning etc. We did not find any articles studying the effect of motivation on learning and academic performance. Our study therefore adds to the literature on this aspect in medical education. We have also compared subgroups such as males with females and students selected through a qualitative selection procedure with weighted lottery selection, which has never been done before.

**Fig. 2:**
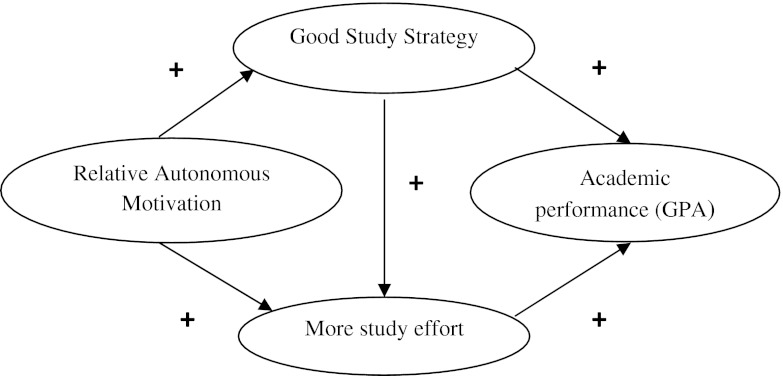
Hypothesized model for motivation influences performance

If there is a priori hypothesis, Structural Equation Modelling (SEM) can employed in research reliably for testing the relationships of different variables with each other, though causality cannot be inferred unless it is an experimental study (Violato and Hecker 2007; Kline 2011). The foundation of a good SEM analyses is a well-founded theoretical basis for relationships being tested in the model (Violato and Hecker 2007; Kline 2011). We had hypotheses, including the directionality of relationships, well-founded in SDT literature. The variables we used in our SEM analyses were Relative Autonomous Motivation (RAM), Good Study Strategy (GSS), Study effort and Academic Performance (see Fig. 2). RAM meant how much of the student’s motivation originated from within himself or herself (autonomous) as compared to that originating from external factors (controlled; Vansteenkiste et al. 2005). GSS meant how much the students studied to understand the study material as against memorizing it without understanding (Biggs et al. 2001). Study effort meant how many hours the student devoted to self-study. Academic Performance meant how the student performed in terms of grades during his medical study.

Our hypotheses were:

- A relative autonomous or self-determined motivation leads to a good study strategy and high study effort, which leads to better academic performance, i.e. the study strategy mediates the influence of motivation on academic performance.
- The overall process and direction of effects are similar among males, females and students admitted through qualitative selection procedure or weighted lottery selection, the relative influence of different factors being different.

We also wanted to study the difference between how the model works in males and females as it has been found before that males have higher CM and lower AM than females (Vansteenkiste et al. 2009; Ratelle et al. 2007; Sobral 2004; Kusurkar et al. submitted). The Netherlands uses a weighted lottery selection system for admitting most students to medical study, and qualitative selection at some schools for a minority of the students. The high school exam grade point average (GPA) of the applicants is weighted according to the score, i.e. the higher the score, the more number of times the student is entered into the lottery, thus giving him or her a higher chance to get selected (Ten Cate 2007). In addition, we wanted to study the difference between how the model works in students selected through qualitative selection and weighted lottery, as students selected through qualitative selection have been found to have higher motivation than those selected through weighted lottery (Hulsman et al. 2007). We therefore designed the present study to test the following research questions and model (see Fig. 2):

1. Does relative autonomous motivation positively affect good study strategy used by students and the study effort?
2. Do good study strategy and high study effort positively affect academic performance?
3. Does this model (Fig. 2) work differently in male and female students? If yes, what are the differences?
4. Does this model work (Fig. 2) differently in students selected through qualitative selection and weighted lottery? If yes, what are the differences?

## Methods

### Sample

Students from years 2 to 6 of the VU University Medical Center Amsterdam were invited to participate in our research project through an electronic questionnaire in September 2009. The thumb rule for a good sample size for SEM is more than 200, a more accurate estimation being 20 subjects for every variable in the model. Our sample size satisfied both rules (Violato and Hecker 2007; Kline 2011).

### Instruments used

The electronic survey designed included some personal data questions, the Academic Motivation Scale (AMS—Cronbach’s alpha ranging from 0.63 to 0.86 for different subscales; Vallerand et al. 1992, 1993) to measure the quality of motivation of the students as described by SDT and the Revised Study Process Questionnaire-2 Factors (R-SPQ-2F—Cronbach’s alpha ranging from 0.57 to 0.72; Biggs et al. 2001) to measure the study strategies used by the students. Academic performance was collected in the form of GPA and European credits (ECs) obtained according to European Credit Transfer System.

### Variables

#### Motivational variables

We used the variables Autonomous Motivation (AM), Controlled Motivation (CM) and Relative Autonomous Motivation (RAM). AM was a measure of the amount of self-determined motivation meaning the motivation which came from within the student. AM was calculated by summing up the average scores on intrinsic motivation and identified regulation subscales of the AMS. CM was a measure of motivation which originated outside of the individual, meaning that it was determined by external factors or reasons. CM was calculated by summing up the average scores on introjected and external regulation subscales of the AMS. Since AM and CM exist simultaneously within an individual, we wanted to create a single score on the relative self-determined motivation, which is put forth as the optimal type of motivation by SDT. RAM was calculated to get a single variable of motivation which incorporated both AM and CM in order to get an idea of the overall self-determined or autonomous motivation. It was calculated by assigning weights to intrinsic motivation (+2), identified regulation (+1), introjected regulation (−1) and external regulation (−2), depending on the placement of this type of motivation in the SDT continuum (see Fig. 1) and summing these weighted scores (Vansteenkiste et al. 2005). AM and CM (Cronbach’s alpha 0.87 and 0.72 respectively) have been reliably and successfully used in earlier studies (Vansteenkiste et al. 2005).

#### Study process related variables

R-SPQ-2F was used to obtain the scores on the strategies used by students when they studied, Deep Strategy (DS) and Surface Strategy (SS; Biggs et al. 2001). Deep Strategy means the strategy used by the student to “maximise meaning” in the material learnt and Surface Strategy means use of rote learning or memorisation of facts (Biggs et al. 2001). Every student employs both types of strategies from time to time. We wanted to use a score which measured relative use of deep strategy, which is considered the “good” type of strategy to be used by students (Biggs et al. 2001). We therefore converted these two scores into a single score called Good Study Strategy (GSS) subtracting the mean SS item scores from the mean DS item scores. A similar type of calculation has been used by Vansteenkiste et al. to create an optimal learning composite from the scores on the LASSI (Learning And Study Strategies Inventory; Vansteenkiste et al. 2005). We also collected self-reported data on study effort (how many hours the student devoted to self-study) among the students.

#### Academic performance variables

All course results obtained by the participating students during one semester (September 2009-January 2010) were obtained from the student information systems of the medical school. To calculate the European credits (ECs), credits from all courses passed within the semester within the programme of medicine were summed. The maximum number of credits that could be obtained per course was the same for each student as per the university rules. Extracurricular credits were not included.

The GPA was defined as the average grade per course, weighted for credits earned (ECs). In the present study only final passing grades were used (grade by which the student had earned credits and passed the course). Within this only courses with a numerical (1–10) final grade were included and only the highest (passing) grade was used to calculate GPA. This was done because we considered the highest grade more important than the attempt at which this grade was obtained, especially since these attempts were made within a very short period of time. Also initial fail grades are not retained in the school’s administration as soon as a pass grade is entered after retake of an exam. Persistent efforts towards learning are as driven by motivation as best performance.

The GPAs of the students from years 2 and 3 were mainly based on courses with cognitive assessments or knowledge tests, whereas GPAs from the students from years 4, 5 and 6 mostly include courses with a mixture of cognitive and knowledge assessments and clinical performance appraisals. These scores were converted into z-scores within the respective groups to make them comparable. We did not assess cognitive and clinical performances separately as clinical performance grades are only available for the subgroup of students in the last phase of the medical study programme.

### Statistical analyses

The software programme SPSS version 15.0 was used for our basic analyses. After checking for normal distribution of the data, linearity of relationships between variables and computing the basic correlations between the different variables, reliability tests for all the scales used to measure the different variables were performed. Multiple regression analysis was planned to determine whether age, gender, year of curriculum and method of admission affected the motivational variables and to compare the model between the groups among whom we found significant effects. Scores on all the variables were converted to z-scores to make them comparable. To compare scores on all variables between subgroups, males and females, and students selected through qualitative selection and weighted lottery, student’s unpaired t-tests were performed.

Structural Equation Modelling (SEM) analysis was carried out using the software AMOS version 5.0. Comparison was done between the proposed and tested model for males and females and for students selected through qualitative selection and weighted lottery. The indices used for estimating goodness of fit of the model were Chi-square goodness of fit value >0.05, Comparison of Fit Index (CFI > 0.09) and Root Mean Square Error of Approximation (RMSEA < 0.05). The acceptable values for a good fit of the model are given in parentheses following each index (Violato and Hecker 2007; Kline 2011).

## Results

The response rate of the students was 26.6% (464/1,742), which included 27.8% (129/464) males and 72.2% (335/464) females. The gender distribution was almost the same as compared to that in the normal medical student population. Students admitted through a weighted lottery selection procedure comprised 81.25% (377/464) and students had been admitted through a qualitative selection procedure comprised 18.75% (87/464). These percentages broadly correspond to the percentage of these students in the whole medical student population, so we consider the sample representative.

We performed analyses on the 383 students as the GPAs of 81 students could not be computed because these students were in an in-between phase in their study where they had completed the previous year, but could not start or finish enough exams to obtain GPAs in the first semester of 2009–2010. The gender and selection distribution characteristics were similar to the overall population, so excluding these students did not adversely affect our results.

The mean age for both, males and females, was 23.3 years (the range was 18–40 years).

The reliabilities of the scales used, i.e. Cronbach’s alphas (see Table 1), ranged from 0.568 to 0.745, which were in line with those found in other studies (Biggs et al. 2001).

**Table 1:** Reliabilities of different scales used

Questionnaire used	Variables	Cronbach’s alpha
AMS	IM	0.800
	IM to know	0.778
	IM accomplishment	0.759
	IM stimulation	0.777
	EM-identified regulation	0.631
	EM-introjected regulation	0.828
	EM-external regulation	0.807
	Amotivation	0.833
	AM	0.745
	CM	0.737
R-SPQ-2F	DS	0.708
	SS	0.568
	GSS	0.621

The correlations between the different variables were as follows (see Table 2): AM and CM were significantly positively correlated which was expected as it had been observed in earlier studies (Vansteenkiste et al. 2005). RAM was significantly positively correlated with AM and significantly negatively correlated with CM, which showed that the computation of RAM as a variable was well-founded. AM and RAM were significantly negatively correlated with amotivation. RAM was significantly positively correlated with Good Study Strategy and GPA. These correlations formed the basis for testing the model proposed in Fig. 2.

**Table 2:** Pearson correlations between the variables of all students (n = 383)

Variables	AM	CM	RAM	Amotivation	Good SS	Study effort	GPA
AM	–						
CM	0.409**	–					
RAM	0.240**	−0.764**	–				
Amotivation	−0.305**	0.068	−.0269**	–			
Good SS	0.384**	−0.041	0.352**	−0.313**	–		
Study effort	0.091	0.029	0.042	−0.088	0.231**	–	
GPA	0.147**	−0.009	0.121*	−0.097	0.218**	0.137**	–
ECs	−0.062	−0.006	0.050	0.027	0.108*	−0.153**	0.158**

*AM* autonomous motivation, *CM* controlled motivation, *RAM* relative autonomous motivation, *SS* study strategy, *GPA* grade point average, *EC* European credits

* *p* < 0.05; ** *p* < 0.01; *** *p* < 0.001

A regression analysis was performed to find out whether age, gender, year of curriculum and method of admission (qualitative versus weighted lottery selection) affected RAM and it was found that the effects of gender (R^2^ = 0.046, *p* = 0.000) and method of admission (R^2^ = 0.015, *p* = 0.009) were significant and the effects of age (*p* = 0.071) and year of curriculum (*p* = 0.368) were not significant.

Since gender had a significant effect on RAM, we performed student’s unpaired t-test to compare males with females. We found that males had significantly higher CM, significantly lower RAM and significantly lower GPAs as compared to the females (see Table 3).

**Table 3:** Results of T test comparing Males (n = 110) with Females (n = 273) and students selected through weighted lottery (n = 318) with those selected through a Qualitative selection procedure (n = 65)

Variable	Males (mean ± SD)	Females (mean ± SD)	95% CI between z-scores of means	*p* value for difference between z-scores
AM	5.309 ± 0.80	5.353 ± 0.67	−0.287, 0.161	0.582
CM	4.464 ± 1.11	3.996 ± 1.13	0.189, 0.628	0.000***
RAM	2.369 ± 2.97	4.047 ± 3.51	−0.717, −0.274	0.000***
Amotivation	1.490 ± 0.71	1.459 ± 0.78	−0.178, 0.258	0.719
Good SS	5.418 ± 0.96	5.559 ± 0.95	−0.362, 0.073	0.194
Study effort	14.399 ± 8.19	14.872 ± 7.38	−0.284, 0.161	0.586
ECs	22.247 ± 10.14	23.467 ± 9.09	−0.299, 0.078	0.252
GPA	7.177 ± 0.72	7.367 ± 0.79	−0.362, −0.016	0.000***

	Weighted lottery selection (mean ± SD)	Qualitative selection procedure (mean ± SD)	95% CI between z-scores of means	*p* value for difference between z-scores
AM	5.298 ± 0.719	5.548 ± 0.656	−0.620, −0.083	0.010*
CM	4.143 ± 1.14	4.069 ± 1.19	−0.204, 0.333	0.636
RAM	3.357 ± 3.42	4.584 ± 3.51	−0.633, −0.091	0.009**
Amotivation	1.507 ± 0.80	1.280 ± 0.52	0.028, 0.551	0.005**
Good SS	5.485 ± 0.97	5.690 ± 0.87	−0.475, 0.057	0.124
Study effort	14.805 ± 7.84	14.390 ± 6.46	−0.214, 0.323	0.654
ECs	23.64 ± 9.86	20.538 ± 6.24	0.110, 0.451	0.001**
GPA	7.40 ± 0.757	6.84 ± 0.733	0.364, 0.767	0.657

* *p* < 0.05; ** *p* < 0.01; *** *p* < 0.001

Since method of admission significantly affected RAM we performed student’s unpaired t-test to compare students selected through qualitative selection and weighted lottery. We found that students selected through qualitative selection had significantly higher AM and RAM and significantly lower ECs and amotivation as compared to those admitted through weighted lottery (see Table 3).

**Fig. 3:**
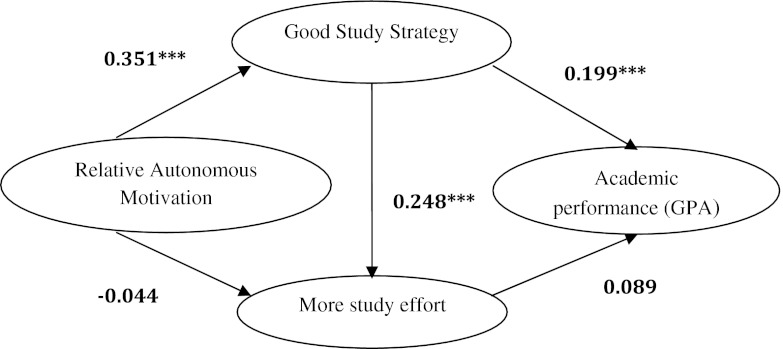
Structural equation model depicting relationship between motivation, study strategy and performance for all students. ***p < 0.001

The structural equation model analyses, which included comparing the male and female groups, resulted in the model depicted in Fig. 3 and had the following characteristics: n = 383, *df* = 3, Chi square = 1.095, *p* = 0.778 (>0.05) i.e. non-significant (Chi-square goodness of fit), so this model was a good model. CFI = 1.000 (>0.09), RMSEA (Root Mean Square Error of Approximation) model fit was equal to 0.000 (<0.05), which was a good fit. The model fit both male and female groups very well (characteristics of the models remained the same as mentioned above), but the regression weights for the different relationships between both groups were different (see Table 4).

**Table 4:** Differences in regression weights of variables between models for all, males, females, qualitative selection and lottery selection

Variables	Model				
	Males	Females	Qualitative selection	Weighted lottery selection	All
RAM on Good SS	0.321***	0.355***	0.324**	0.349***	0.351***
RAM on study effort	−0.118	−0.019	−0.121	−0.025	−0.044
Good SS on study effort	0.288**	0.227***	0.306*	0.240***	0.248***
Good SS on GPA	0.007	0.260***	0.319**	0.178**	0.199***
Study effort on GPA	0.205*	0.045	0.222^Ŧ^	0.063	0.089

* *p* < 0.05; ** *p* < 0.01; *** *p* < 0.001; ^Ŧ^ *p* = 0.059

We used the same model for the structural equation model analyses which included comparing the qualitative and weighted lottery selection subgroups and the model (See Fig. 3) had the following characteristics: n = 383, Chi square = 4.709, *df* = 3, *p* = 0.194 (>0.50), therefore non-significant (Chi-square goodness of fit). The CFI = 0.990 (>0.09) and RMSEA model fit for this was 0.027 (<0.05), which is a good fit. The model fit both qualitative and weighted lottery selection subgroups very well (characteristics of the models remained the same as mentioned above), but the regression weights for the different relationships between both groups were different (see Table 4).

We found in the present study that relative autonomous motivation is positively associated with the use of a good study strategy by the students, which is positively associated with high study effort and better GPA (see Fig. 3; Table 4). The relative associations for these relationships were different in males, females, qualitative and weighted lottery selection subgroups (see Table 4).

## Discussion

In the present study, we found that relative autonomous motivation is positively associated with the use of a good study strategy by the students which is positively associated with higher study effort (also found by Wilkinson 2007, though not through SEM analyses; Wilkinson et al. 2007) and better GPA. Vansteenkiste et al. found that a similar model incorporating self-study hours within the variable “Optimal Learning composite” fit students in the same age group (mean age about 23 years) in a general education study well (Vansteenkiste et al. 2005). Relative autonomous motivation is significantly associated with higher GPA (also found by Sobral 2004), but the relation seems to be more indirect, i.e. through use of good study strategy, instead of a direct relation. The positive correlation of autonomous motivation with deep study strategy (Sobral 2004) and deep study strategy with academic performance is supported by in other studies in medical education (Sobral 2004; McManus et al. 1998). We had expected to find significant positive association between relative autonomous motivation and study effort, but did not find this.

As expected, differences were found in the nature of relationships between males and females and qualitative and weighted lottery selection subgroups. Significant positive association of RAM on GSS and GSS on high study effort were found in all the four groups, so these relationships seem to be well-substantiated. With the exception of the males subgroup, GSS showed significant positive association with GPA in all subgroups. Study effort showed significant positive association with GPA only in the males and qualitative selection subgroups, and no significant positive association in the overall model. This means that some variables have stronger associations in some subgroups and weaker associations in others. RAM seems to have an indirect positive association with GPA (Pearson correlation = 0.121, *p* < 0.001) through its positive association with GSS, rather than having a direct association, in all except the males subgroup. In the qualitative selection subgroup, RAM seems to have an indirect positive association with GPA (Pearson correlation = 0.121, *p* < 0.001) also through its positive association with study effort. One of the criteria for admission through a qualitative selection was evidence of significant time investment in certain eligible activities parallel to full time education, including health-related work, of at least 160 h per year. Our finding that this subgroup showed higher association of study effort with GPA serves as a validation for our findings. In the males subgroup, RAM seems to have an indirect positive association with GPA (Pearson correlation = 0.121, *p* < 0.001) only through its positive association with study effort.

Vansteenkiste et al. found differences in the scores of males and females on some variables, but did not compare their proposed model between male and female groups (Vansteenkiste et al. 2005). Our study thus adds to Vansteenkiste et al. study.

Findings of other studies in medical education support the differences found between males and females in the present study in the quantity of motivation (Females > Males; Kusurkar et al. 2010; Carlo et al. 2003; Loucks et al. 1979) and quality of motivation (females have higher autonomous or intrinsic and lower controlled or extrinsic motivation than males; Kusurkar, Croiset and Ten Cate (submitted); Buddeberg-Fischer et al. 2003). The differences between the qualitative and weighted lottery selection subgroups in the quantity of motivation (Qualitative selection > Weighted lottery selection) are also supported by similar findings in other studies (Hulsman et al. 2007). Thus, our study also adds to the study of differences between medical students selected by qualitative and weighted lottery selection.

Thus we found acceptable evidence for our proposed model which was based on a priori hypothesis derived from SDT.

### Implications

Our study provides acceptable evidence that the quality of motivation is important in determining good performance among medical students through good study strategy and high effort. These findings imply that we should specifically attempt to target enhancing autonomous motivation among medical students in order to encourage an attitude towards deep learning and high effort and finally good performance.

### Strengths and limitations

One of the strengths of our study is that we used a structural equation modelling approach and have found a well-fitting model for the relationship between motivation, study strategy, effort and academic performance. Another strength is that we have compared the model between male and female subgroups and qualitative and weighted lottery selection subgroups. Since this study was performed in the Netherlands we were in a unique position to compare the latter two subgroups.

The major limitation of our study was a low response rate. However, given the fact that the responding population seemed representative of the medical student population in general, and that the absolute number of responses allows for structural equation analysis, we consider reporting our findings to add to the existing literature. The other is that this study was carried out at a single university in the Netherlands and hence has limited generalisability. It can very well serve as a good starting point for more studies on the same aspect in medical education.

## Conclusion

Relative Autonomous Motivation positively affects academic performance through deep strategy towards study and higher study effort. This model seems valid in medical education in subgroups such as males, females, students selected by qualitative and weighted lottery selection procedures.
